# RNA-Seq Revealed a Circular RNA-microRNA-mRNA Regulatory Network in Hantaan Virus Infection

**DOI:** 10.3389/fcimb.2020.00097

**Published:** 2020-03-13

**Authors:** Shuang Lu, Ni Zhu, Weiwei Guo, Xin Wang, Kaiji Li, Jie Yan, Cuiping Jiang, Shiyu Han, Hanmin Xiang, Xiaohan Wu, Yuanyuan Liu, Hairong Xiong, Liangjun Chen, Zuojiong Gong, Fan Luo, Wei Hou

**Affiliations:** ^1^State Key Laboratory of Virology, Institute of Medical Virology, School of Basic Medical Sciences and Department of Infectious Diseases, Renmin Hospital, Wuhan University, Wuhan, China; ^2^Department of Microbiology, School of Basic Medical Sciences, Hubei University of Science and Technology, Xianning, China

**Keywords:** HTNV, circular RNA, microRNA, competing endogenous RNA network, cellular response changes, viral replication

## Abstract

Hantaan virus (HTNV), a Hantavirus serotype that is prevalent in Asia, causes hemorrhagic fever with renal syndrome (HFRS) with high mortality in human race. However, the pathogenesis of HTNV infection remains elusive. Circular RNAs (circRNAs), a new type of non-coding RNAs, play a crucial role in various pathogenic processes. Nevertheless, circRNA expression profiles and their effects on pathogenesis of HTNV infection are still completely unknown. In the present study, RNA sequencing was performed to analyze the circRNA, microRNA (miRNA), and mRNA expression profiles in HTNV-infected and mock-infected human umbilical vein endothelial cells (HUVECs). A total of 70 circRNAs, 66 miRNAs, and 788 mRNAs were differently expressed. Several differentially expressed RNAs were validated by RT-qPCR. Moreover, we verified that some differentially expressed RNAs, such as circ_0000479, miR-149-5p, miR-330-5p, miR-411-3p, RIG-I, CMPK2, PARP10, and GBP1, promoted or inhibited HTNV replication. Gene Ontology (GO) and Kyoto Encyclopedia of Genes and Genomes (KEGG) enrichment analysis demonstrated that the host genes of differentially expressed circRNAs were principally involved in the innate immune response, the type I interferon (IFN) signaling pathway, and the cytokine-mediated signaling pathway. Additionally, the circRNA-miRNA-mRNA regulatory network was integrally analyzed. The data showed that there were many circRNA-miRNA-mRNA interactions in HTNV infection. By dual-luciferase reporter assay, we confirmed that circ_0000479 indirectly regulated RIG-I expression by sponging miR-149-5p, hampering viral replication. This study for the first time presents a comprehensive overview of circRNAs induced by HTNV and reveals that a network of enriched circRNAs and circRNA-associated competitive endogenous RNAs (ceRNAs) is involved in the regulation of HTNV infection, thus offering new insight into the mechanisms underlying HTNV-host interaction.

**Graphical Abstract:**
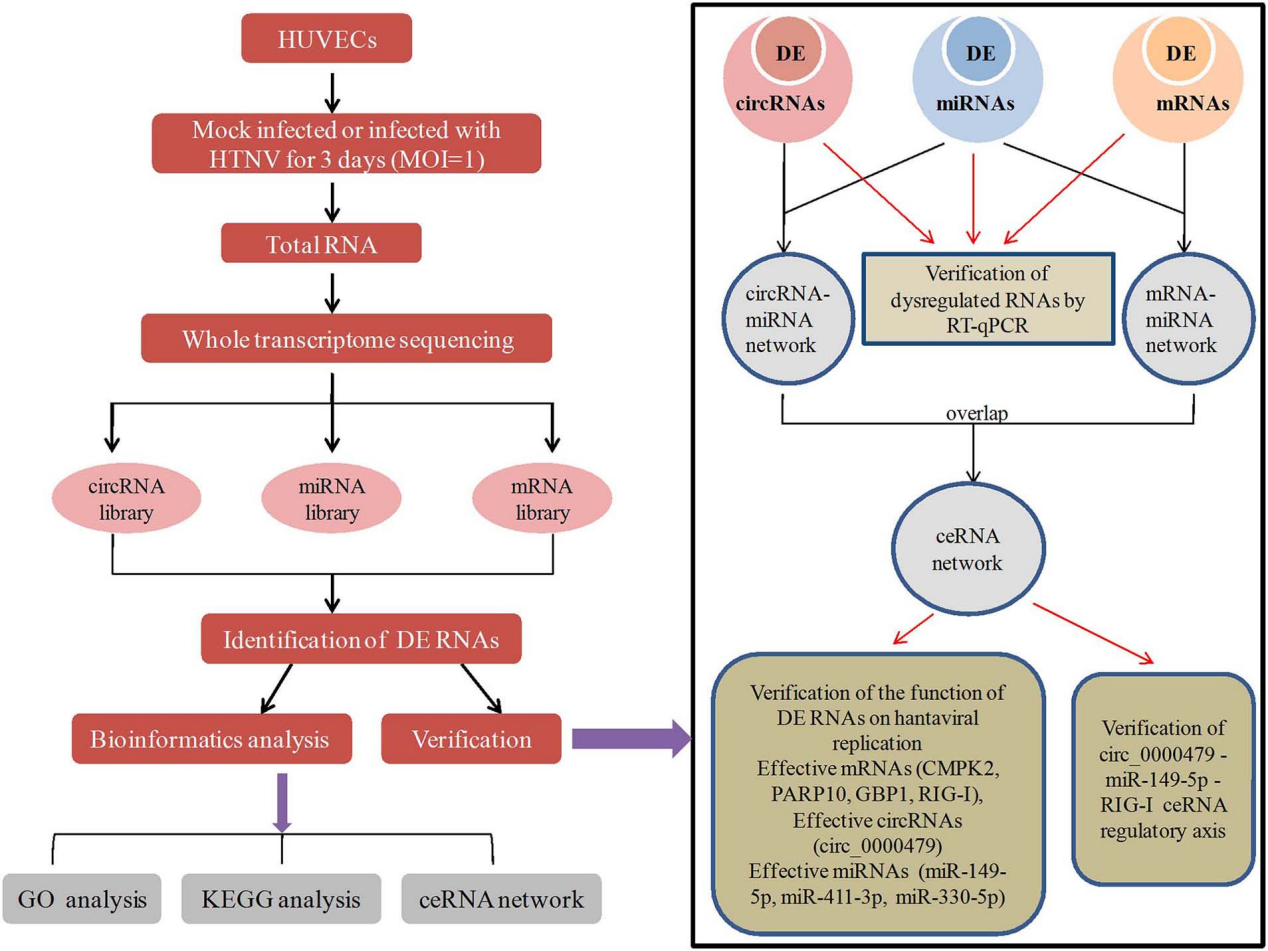
In this work, HUVECs were mock infected or infected with HTNV for 3 days. Total RNA of the cells were analyzed by RNA-seq and obtained circRNA, miRNA, mRNA library. Differentially expressed (DE) RNAs were identified and subjected to GO analysis, KEGG analysis and ceRNA network construction. Then 8 DE circRNAs, 8 DE mRNAs, 6 DE miRNAs were verified by RT-qPCR. Besides, mRNAs (CMPK2, PARP10, GBP1, and RIG-I), circRNAs (circ_0000479), miRNAs (miR-149-5p, miR-411-3p, and miR-330-5p) in the ceRNA network were found effective to inhibit or promote virus replication. And the circ_0000479-miR-149-5p-RIG-I ceRNA axis was verified in HTNV infection.

## Introduction

Hantaviruses, which belong to the class *Bunyavirales*, order *Hantaviridae*, are widespread reemerging zoonotic pathogens. The hantaviral genome consists of three negative sense single-strand RNA segments, designated as S, M, and L, encoding nucleocapsid protein (NP), glycoprotein precursor (GPC), and viral RNA-dependent RNA polymerase (RdRp), respectively (Kariwa et al., 2007). Pathogenic hantaviruses infection in humans mainly causes hemorrhagic fever with renal syndrome (HFRS), hantavirus pulmonary syndrome (HPS) or hantavirus cardiopulmonary syndrome (HCPS), and a generally milder form of HFRS, which is also called nephropathia epidemica (NE) (Echterdiek et al., 2019). In China, HFRS is mainly caused by Hantaan virus (HTNV) and Seoul virus (SEOV) and the incidence and mortality of HFRS are highest of the world (Zheng et al., 2018). Research has shown that hantavirus mainly infects human vascular endothelial cells, causing destruction of capillary integrity, and altering vascular permeability (Jiang et al., 2016). Moreover, increased capillary leakage is the basic pathogenic feature for both HFRS and HCPS (Terajima and Ennis, 2011). Rapidly growing evidence is now mounting that there is a considerable overlap of clinical presentations between HFRS and HPS (Clement et al., 2014). The temporary capillary leak results in sometimes massive interstitial generalized fluid accumulation, leading to different degrees of multiple organ failure (MOF), mainly expressed as acute kidney injury (AKI), and/or as acute lung edema, with or without circulatory shock (Macneil et al., 2011; Jiang et al., 2016; Garanina et al., 2019). However, hantavirus is not a lytic virus. These findings strongly imply that hantavirus infection should regulate host cells function to coordinate viral propagation, but the association between HTNV-host cells interaction and HTNV pathogenesis has not been comprehensively studied.

As much as 80% of the host genome is transcribed to non-translated RNA, which underscore the potential importance of host non-coding RNA (ncRNA) in the host-virus interaction. Accumulating evidence shows that host ncRNAs play key regulatory roles during viral replication and infection (Lamontagne et al., 2015; Ding et al., 2016; Landeras-Bueno and Ortin, 2016; Liu and Ding, 2017; Trobaugh and Klimstra, 2017; Tao et al., 2018). To date, few studies have been published about the functions of ncRNAs in HTNV infection. It was reported that the long non-coding RNA (lncRNA) NEAT1 controls HTNV infection by promoting RIG-I and DDX60 expression and interferon (IFN) responses (Ma et al., 2017). LncRNA NRIR facilitates HTNV infection by negatively regulating the transcription of IFITM3 (Xu-Yang et al., 2016). MicroRNA (miRNA) expression profiles have been studied in HTNV-infected A172 cells, but without a focus on the functions and mechanisms involved in the regulation of viral infection (Shin et al., 2014). Besides, our group demonstrated that miR-146a can negatively regulate the release of HTNV-induced pro-inflammatory cytokines to promote viral replication (Chen Q. Z. et al., 2017).

Additional to lncRNAs and miRNAs, circular RNAs (circRNAs) are a new category of endogenous ncRNA (Qu et al., 2015). The majority of circRNAs regulate the gene expression by sponging miRNA, serving as competing endogenous RNA (ceRNA) (Hansen et al., 2013; Panda, 2018); this phenomenon has been identified in many diseases (Wang M. et al., 2018; Mao et al., 2019; Rong et al., 2019). More importantly, recent studies have found that circRNAs have substantial effects in host-virus interactions. For the integrative analysis of circRNAs, miRNAs and genes, it was found that the differentially expressed circRNAs were involved in cellular responses to Herpes Simplex Virus 1, human cytomegalovirus, and HIV infection through the circRNA-miRNA-gene regulatory axis. Moreover, these genes regulated by circRNAs are enriched in inflammatory response, defense response to virus, and pathways of apoptosis, which may account for viral pathogenesis and cellular immunity (Shi et al., 2018; Zhang et al., 2018; Lou et al., 2019). Artificial circRNAs inhibit the production of HCV viral proteins by effectively adsorbing cellular miR-122 (Jost et al., 2018), and the delivery of purified circRNAs can activate RIG-I-mediated immune responses and provides effective protection against viral infection (Chen Y. G. et al., 2017). Upon viral infection, the nuclear export of NF90/NF110 to the cytoplasm can decrease the expression of circRNA, and the dissociation of NF90/NF110 from circRNPs can inhibit viral replication (Li et al., 2017). However, so far, the specific characteristics and roles of circRNAs and circRNA-associated ceRNAs in HTNV infection have not yet been reported.

Herein, we investigated and analyzed the profiles of circRNAs expression and the potential roles of circRNAs in HTNV-host interaction through intergrated omics data of RNA sequencing and miRNA sequencing on mock-infected and HTNV-infected HUVECs. The identified network of circRNAs and circRNA-associated ceRNAs could reveal the complex regulatory function of circRNAs in the interplay between HTNV and host. In summary, our results provide novel insight regarding the roles of circRNAs in HTNV infection and broaden our understanding of the mechanisms underlying HTNV pathogenesis.

## Materials and Methods

### Cell Culture and Viral Infection

HUVECs were purchased from ScienCell (Cat No. 8000, Carlsbad, USA) and cultured in endothelial cell medium (ECM) with 5% fetal bovine serum, 1% penicillin-streptomycin, and 1% endothelial cell growth supplement (ScienCell) at 37°C and 5% CO_2_. HEK293T and Vero E6 cells were purchased from the American Type Culture Collection (ATCC) and cultured in Dulbecco's modified Eagle's medium (DMEM) supplemented with 10% FBS (Gibco) at 37°C and 5% CO_2_. The HTNV 76-118 strain was obtained from the Institute of Virology, Chinese Center for Disease Control and Prevention (CDC, Beijing, China) and propagated in Vero E6 cells. HUVECs were mock-infected or HTNV-infected at a multiplicity of infection (MOI) of 1.

### RNA Extraction, RNAs Libraries Construction

mRNA library construction: At 72 h post-infection, total RNA was extracted from mock-infected and HTNV-infected HUVECs with TRIzol (Invitrogen, USA), followed by the assessment of concentration, purity, and integrity using the ND-1000 Nanodrop (Thermo Fisher, USA) and Agilent 2200 TapeStation (Agilent Technologies, USA). Ribosomal RNA (rRNA) was removed from total RNA using an Epicenter Ribo-Zero rRNA Removal Kit (illumina, USA) and RNA was fragmented to ~200 bp. Single stranded cDNA was synthesized by reverse transcription, which was followed by double-stranded cDNA synthesis. Subsequently, the fragments were ligated and purified, and PCR was performed to amplify sequences. RNA was optimized and purified using the NEBNext® Ultra™ Directional RNA Library Prep Kit for Illumina (NEB, USA). The Agilent 2200 TapeStation and Qubit®2.0 (Life Technologies, USA) was employed to evaluated the purified library products. The quality-tested RNA was sequenced on a HiSeq3000 (Illumina) using the pair-end flow cell, and the mRNA library was obtained.

CircRNA libraries were constructed as follows: Total RNA was isolated from HUVECs by the TRIzol Reagent (Invitrogen) according to the manufacturer's protocol. RNA purity was assessed by ND-1000 Nanodrop with a criteria of A260/280 ≥1.8 and A260/A230 ≥2.0. RNA integrity (RIN) was evaluated by Agilent 2200 TapeStation (Agilent Technologies, USA) requiring RIN ≥7.0. After that, ~2 μg of total RNA from each sample was subjected to the Epicenter Ribo-Zero rRNA Removal Kit (illumina, USA) to remove ribosomal RNA, followed by incubation with 40 U RNase R for 3 h at 37°C (Epicenter, USA) to remove linear RNA. Subsequently, the purified RNA were fragmented to ~200 bp and were subjected to first strand and second strand cDNA synthesis, and adaptor ligation. CircRNA libraries were prepared by using NEBNext® Ultra™ RNA Library Prep Kit for Illumina (NEB, USA) according to instructions. The Agilent 2200 TapeStation and Qubit®2.0 (Life Technologies, USA) was employed to control the purity of library products and then sequenced on HiSeq 3000 (Illumina) with 2 × 150 bp mode (Memczak et al., 2013). Small RNA libraries were constructed as follows: Total RNA was isolated, and the purity and integrity were evaluated as mentioned above. Each sample had a RIN^e^ (RNA integrity number) value >7.0. In brief, RNAs successively were ligated with the 3′RNA adapter and 5′ adapter. Subsequently, the ligated RNAs were performed to RT-PCR and amplification. Then the PCR products of 18–40 nt were selected by polyacrylamide gel electrophoresis (PAGE) gel according to instructions of the NEBNext® Multiplex Small RNA Library Prep Set for Illumina® (Illumina, USA). Thereafter, the purified library products were evaluated using the Agilent 2200 TapeStation and sequenced on HiSeq2500 platform.

### Quantification and Differential Expression Analysis of circRNAs, miRNAs, and mRNAs

#### Identification and Quantification of circRNAs

As shown in Figure S1A, the raw data obtained from RNA sequencing were first filtered by Trimmomatic-0.36 program to remove adapter sequences, invalid reads and low quality reads. Afterwards, rRNA residues were removed by comparing known rRNA information in RNA central database to gain clean reads. Clean reads were then mapped to the human reference genome (hg19) from UCSC genome database (http://genome.ucsc.edu/) by Tophat (v2.0.13) program. The unmapped reads were performed fusion gene alignment using Tophat-Fusion (v2.0.13) to identify candidate circRNAs (Zheng et al., 2016). The criteria were as follows: GT/AG splicing signals, mismatch ≤ 2, back-spliced junction reads ≥1, and distance between two splice sites is no more than 100 kb as previous reported (Shi et al., 2018). The circRNA expression level was described as RPM (Reads Per Million mapped reads). Differential expression analysis of circRNAs were performed with edgeR package (v3.20.9), and identified by threshold values of |log_2_(fold-change)| >1 and *q* < 0.05.

#### Analysis of mRNAs Expressoin

As shown in Figure S1B, the reads containing adapter sequences, low-quality reads and undetermined bases were cleaned from raw data by Trimmomatic-0.36 program. Then high-quality clean reads were gained through removing rRNA residues. The clean reads were aligned to the reference human genome (hg19) using Tophat (v2.0.13). The RefSeq databases were chosen as the annotation references. The mRNAs expression were calculated as Reads Per Kilobase per Millions reads (RPKM). The differential expression of mRNAs were analyzed with edgeR package (v3.20.9). The criterion of |log_2_(fold-change)| >1 and *q* < 0.05 was used to identify differentially expressed mRNAs.

#### Analysis of miRNAs Expressoin

As shown in Figure S1C, the raw reads obtained from miRNA library were first filtered by Cutadapt 1.8.1. The clean reads were then mapped to hg19 reference genome using BWA v0.7.12 to identify miRNAs. The miRNAs in each group were identified basing on the known miRNAs downloaded from the miRBase database v21.0 (www.mirbase.org) (Wu et al., 2018). The miRNAs expression were calculated by reads per million clean tags (RPM). Differential miRNAs were identified using thresholds: |log_2_(Fold-change)|>1 and *p* < 0.05. RNA-seq data were uploaded into the gene expression omnibus (GEO) database (https://www.ncbi.nlm.nih.gov/geo/) (accession nos. GSE133634, GSE133751, GSE133319).

### GO and KEGG Pathway Enrichment

Gene Ontology (GO) enrichment analysis and Kyoto Encyclopedia of Genes and Genomes (KEGG) pathway analysis were performed to analyze the advanced functions of DE genes, DE miRNA targeted genes, and parental genes of DE circRNAs. GO analysis labels gene with a function, such as molecular function, biological process, or cellular component. KEGG analysis provides annotation information of signal transduction and disease pathways for genes, thus providing a basis for gene function and pathway research. The analyses were based on the GO resource (http://www.geneontology.org), the KEGG database (http://www.genome.jp/kegg/), and KOBAS 2.0 software. A *P* < 0.05 was considered statistically significant enrichment.

### Co-expression Network Analysis

#### Construction of circRNA-miRNA Co-expression Network

Firstly, we selected 8 DE circRNAs verified by real-time quantitative PCR, and then identified miRNAs targeting on these circRNAs using miRanda (http://www.microrna.org/microrna/home.do), TargetScan (http://www.targetscan.org/vert_72/), and RNAhybrid (https://bibiserv.cebitec.uni-bielefeld.de/rnahybrid), which were widely used public databases. The candidate miRNA-circRNA relationships were picked out by thresholds of miRanda (Total Sore ≥ 140; Total Energy < −17 kmol), RNAhybrid (minimum free energy ≤ −21) and Targetscan (conserved groups).

#### Construction of miRNA-mRNA Co-expression Network

TargetScan (http://www.targetscan.org/vert_72/), miRDB (http://www.mirdb.org/), miRTarBase (http://miRTarBase.mbc.nctu.edu.tw/), and miRWalk (http://zmf.umm.uni-heidelberg.de/apps/zmf/mirwalk2/) were applied to predict the target DE mRNAs of miRNAs of circRNA-miRNA network. The intersection of mRNAs predicted by at least 2 databases was used to establish miRNA-mRNA network.

#### ceRNA Network Analysis

In the constructed miRNA-mRNA network, we selected 80 mRNAs enriched in the innate immune pathway based on the GO and KEGG analyses as candidates. These mRNAs were then confirmed to predict the miRNA-mRNA pairs. Next, circRNA-miRNA pairs sharing the same miRNAs with above miRNA-mRNA pairs were identified as candidate circRNA-miRNA-mRNA competing interaction. Pearson correlation coefficient (r) and correlation *P*-value (cP*-*value) were used to estimate the co-expression relationship between circRNAs, mRNAs, and miRNAs. The strong interactions between miRNAs, circRNAs, and mRNAs (with a threshold of r > 0.85 or r < −0.85 and cP-value < 0.05) were selected to establish ceRNA network using Cytoscape (v3.4.0).

### Transfection and Viral Infection

Specific miRNA mimics, mimic control, siRNAs against circRNA and mRNA, and siRNA control were synthesized by Ribobio (Guangzhou, China). The siRNA sequences were listed in Table 1. HUVECs were ~50–80% confluent, followed by transfection with 50 nM of siRNAs or 10 nM of mimics using Highgene Transfection Reagent (ABclonal, China) following the manufacturer's protocol. At 24 h post-transfection, besides detection of uptake efficiency, HUVECs were rinsed three times by PBS and then infected with a certain volume of viral suspension gained a multiplicity of infection (MOI) of 1. After incubation for 2 h, the viral suspension was discarded and replaced with ECM medium to culture for another 72 h.

**Table 1:** Target sequence of siRNAs.

**siRNA**	**Target sequence**
siRIG-I	TTTGTCGCTAATCCGTGAT
siCMPK2_01	CCAGGTTGTTGCCATCGAA
siCMPK2_02	GGAGGAGTGTACCTCCTTT
siCMPK2_03	GGCCTCCGAAATAGCTAAA
siISG15_01	TGCGACGAACCTCTGAGCA
siISG15_02	TCCTGGTGAGGAATAACAA
siISG15_03	GGTGGACAAATGCGACGAA
siPARP10_01	GGTAGAGGGATTATGACAA
siPARP10_02	CTACCATGAGGACCTTCTT
siPARP10_03	GCAGCATTAGCTGCCATGT
siSAMD9_01	GGTTAGACCTTGACAGTGA
siSAMD9_02	GAACAGGTAACCAGTTTAA
siSAMD9_03	GAACCTGACAATTCTTATA
siGBP1_01	AAGGCATGTACCATAAGCT
siGBP1_02	GAGACGACGAAAGGCATGT
siGBP1_03	GGAGTCTATGACTGATGCA
siCirc_0000479	GAGCATCAGCAATACACAAGT
siCirc_0006132	ATACACAAGACCGATGTTGTT
siCirc_0007793	AGGAAGCAAAAGAAGGAAAAA
siCirc_0046034	AGCAGGTGTTGGTCTTCTGCA

### Validation of circRNAs, miRNAs, and mRNAs Expression Using Real-Time Quantitative PCR (RT-qPCR) Analysis

To confirm the results of sequencing, firstly, the simple random sampling method was used in selecting RNAs with random numbers generated in Microsoft EXCEL. Whereafter, total RNAs of HTNV-infected and mock-infected cells were extracted using TRIzol reagent (Invitrogen, USA), and reversely transcribed by reverse transcriptase (M-MLV, Promega) according to the manufacturer's protocol. Then, RT-qPCR was performed with SYBR Green qPCR Master Mix (Bio-Rad, USA) on CFX96 real-time PCR system. The used primers were listed in Table 2. The relative quantification of miRNAs, circRNAs, and mRNAs were normalized to U6 or GAPDH with the 2^−ΔΔ*CT*^ method.

**Table 2:** Primers used for quantitative real time PCR.

**RNA**	**Forward primer**	**Reverse primer**
CMPK2	GCCAACAGTGTGTTTCGTCA	TACCGTCTGCAGGACCTTTTC
CXCL11	TGTCTTTGCATAGGCCCTGG	TGATTATAAGCCTTGCTTGCTTCG
ISG15	GAGAGGCAGCGAACTCATCTT	CCAGCATCTTCACCGTCAGG
USP18	GGCTCCTGAGGCAAATCTGT	CAACCAGGCCATGAGGGTAG
RIG-I	ATGGGACGAAGCAGTATTTAG	GCTTGGGATGTGGTCTACTC
SAMD9	TGGGGGAACTACCTTGGCTA	CGGTTCATTGCCCCATAGGT
GBP1	TTGAGAACACTAATGGGCGACT	TAGGATTTGCCTGTGCGGT
PARP10	GACACAGGCCTTGAAGAGGTG	CCTGGTCACCACCTGTACTGTC
circ_0000479	GAAGCATTTAGAGAGCATCAGCA	GCTCCGCAATTCTTTGTATCTCAT
circ_0002470	CTGATGAGCTGGTCTGCGAT	TCCTTACCCGCATTCTCACC
circ_0006132	TGCACCCGAGTGATCATGAAA	CCCTTGTCCAACTGTGTCCAA
circ_0007793	ACGATCAACGGCGAATGACT	GGGCACAGGGTGAAGATACC
circ_0022228	AGATCTTTGCCAGCATCCCA	GTTGTACAGGACCTCCCCAAT
circ_0046034	TGTTCAATGACCGGCTGTGT	ATCCACGTCCTGAACAGCATA
circ_0066996	CTGCAATGTGGTATGCAGAGTT	TCACACAGCTGACGCTCATT
circ_0073948	GAACGAAACACGCCATCTGC	GGGCTGAGCTGGTATGAGTC

### Western Blotting

As described in our previous reports (Chen Q. Z. et al., 2017), cells were lysed by RIPA. Protein concentration was measured with BCA protein assay kit. Equal amounts of protein were heated at 95°C for 5 min. Protein was separated by SDS-PAGE and transferred onto polyvinylidene fluoride (PVDF) membranes. Subsequently, the PVDF membrane was blocked with 5% BSA for 40 min at room temperature and then incubated with the primary antibody at 4°C overnight. Finally, the signals on the PVDF membrane were visualized. The primary monoclonal anti-HTNV 76-118 strain antibody was purchased from Abnova (MAB5482, Taiwan). GAPDH was used as internal control. The blots were scanned by ImageJ software.

### Luciferase Reporter Assay

The RIG-I binding region (RIG-I-BR) was amplified by primers (forward 5′-TGC CAT TGT TCT CTT TGA CC-3′ and reverse 5′-GCA ACT ACT GTG TCA TGT AC-3′) and cloned into pmir-GLO. Circ_0000479 full length was amplified by primers (forward 5′-CAC AAG TGC ATA CAC CTT GAT AG-3′ and reverse 5′-TAT TGC TGA TGC TCT CTA AAT GC-3′) and cloned into pmir-GLO and pcDNA3.1(+).

To measure relative luciferase activity, 5 nM of miRNA and 1.2 μg/ml of luciferase reporter DNA or 5 nM of miRNA, 0.6 μg/ml of luciferase reporter DNA and 0.6 μg/ml pcDNA3.1 vector DNA were co-transfected into HEK293T cells. After 48 h of transfection, cells were lysed on ice for 15 min before centrifugation. The supernatant was measured using the Promega Dual-Glo luciferase assay system. Firefly luciferase activity was normalized to that of Renilla.

### Statistical Analysis

Data were expressed as mean ± standard deviation (SD) from at least three independent experiments. SPSS 17.0 software was used for statistical analyses. Differences were determined to be statistically significant at values of *P* < 0.05 by Student's *t*-test for two groups, one-way ANOVA analysis for multiple groups. Single, double and triple asterisks, and ns indicate statistical significance (^*^P < 0.05; ^**^P < 0.01; ^***^P < 0.001) and no significance respectively.

## Results

### Identification and Characterization of circRNAs in HTNV-Infected and Mock-Infected HUVECs

**Figure 1:**
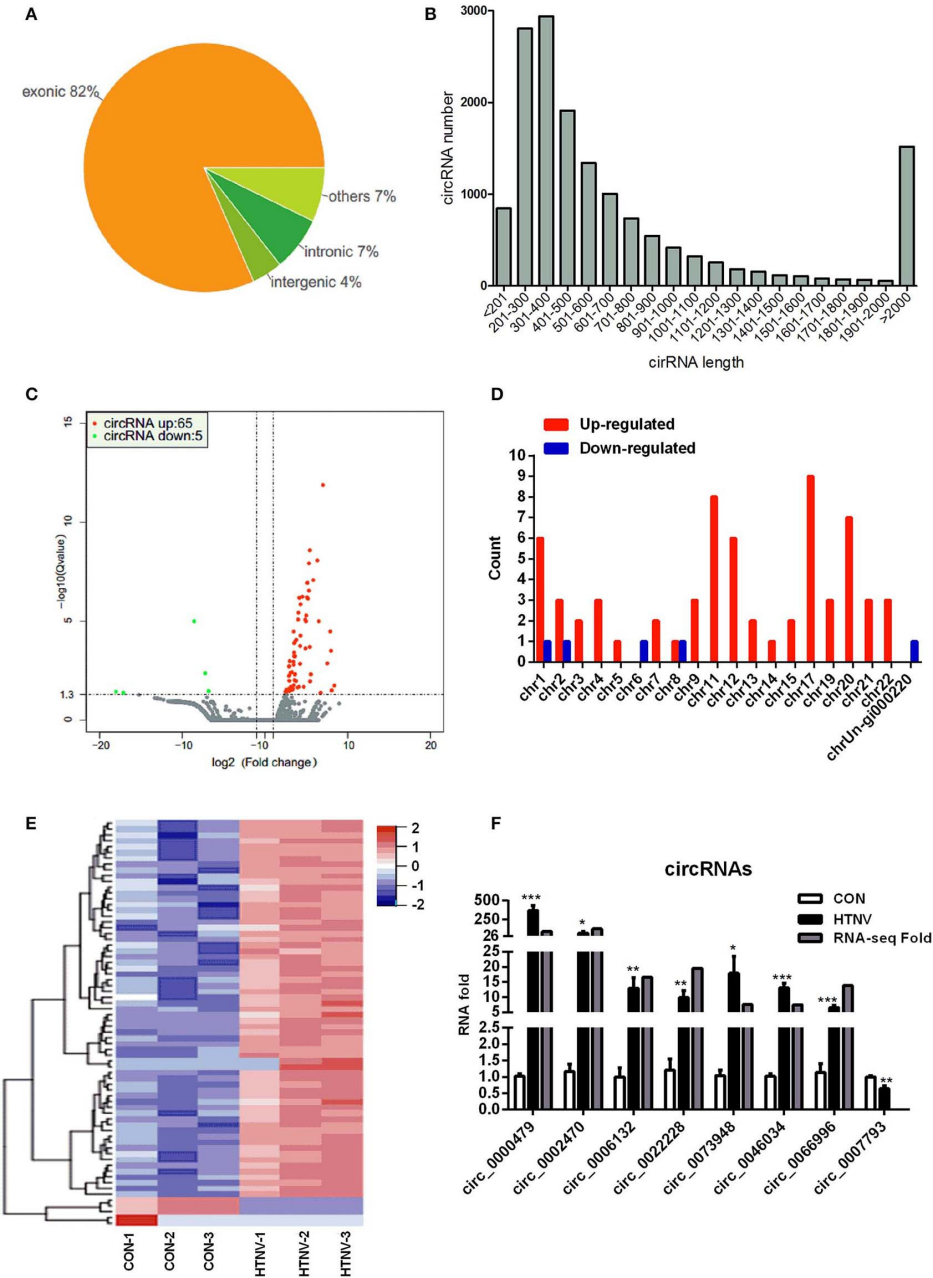
circRNA expression overview. **(A)** circRNAs category chart. **(B)** circRNAs length distribution. **(C)** Volcano plot of DE circRNAs upon HTNV infection in HUVECs. Red dots represent up-regulated circRNAs and green dots represent down-regulated circRNAs. **(D)** Chromosome distribution of DE circRNAs. **(E)** Heatmap and clustering analysis of DE circRNAs. Each row represents one circRNA and each column represents one sample; −2, −1, 0, 1, and 2 represent fold change. Red indicates high expression and blue represents low expression. CON-1, CON-2 and CON-3 represent three mock-infected samples; HTNV-1, HTNV-2, and HTNV-3 represent three HTNV-infected samples. **(F)** Verification of dysregulated circRNAs. HUVECs were infected with HTNV 76-118 (MOI = 1) for 3 days. Then the total RNA was extracted and the expression levels of circRNAs were measured by RT-qPCR. Student's *t*-test, mean ± standard deviation (SD), *P < 0.05; **P < 0.01; ***P < 0.001. The experiment was performed at least three times independently.

We first performed circRNA sequencing, using the rRNA-depleted samples of mock-infected (CON) and HTNV-infected (HTNV) cell cultures. We identified an average of 87 million and 84 million clean reads in the CON and HTNV groups, respectively. The clean reads were used for the identification of circRNAs, and most reads were mapped to the reference genome. The most of circRNAs (accounted for 82%) were composed of exons, while 7% were intronic, and 4% mapped to intergenic regions (Figure 1A). We found that the size of the circRNAs ranged from <200 to >2,000 nt; 81.04% of circRNAs had a predicted spliced length of <1,000 nt; 54.93% had a length <500 nt and 26.11% had a length of 500–1,000 nt (Figure 1B). Based on the filtering criteria (Materials and Methods), a total of 70 DE circRNAs were identified in HTNV-infected samples compared with mock-infected samples (Table S1). Of these, 65 circRNAs were up-regulated and five were down-regulated (Figure 1C). DE circRNAs were widely distributed across chromosomes. Chromosome 1, chromosome 11, chromosome 17, and chromosome 20 contained many up-regulated circRNAs. The five down-regulated circRNAs were distributed on different chromosomes (Figure 1D).

Further, we performed a hierarchical cluster analysis of all DE circRNAs; the results were shown in Figure 1E. Most of the dysregulated circRNAs were lowly expressed in the mock-infected group, but were up-regulated in the HTNV-infected group. The hierarchical cluster analysis classified the expression patterns of mock-infected samples and HTNV-infected samples into different branches (Figure 1E). To confirm the RNA-seq results, we verified the expression of eight randomly selected circRNAs (circ_0000479, circ_0002470, circ_0006132, circ_0007793, circ_0022228, circ_0046034, circ_0066996, and circ_0073948) by RT-qPCR. As shown in Figure 1F, the RT-qPCR results were highly consistent with the RNA-seq results.

### Functional Analysis of the Parental Genes of DE circRNAs

**Figure 2:**
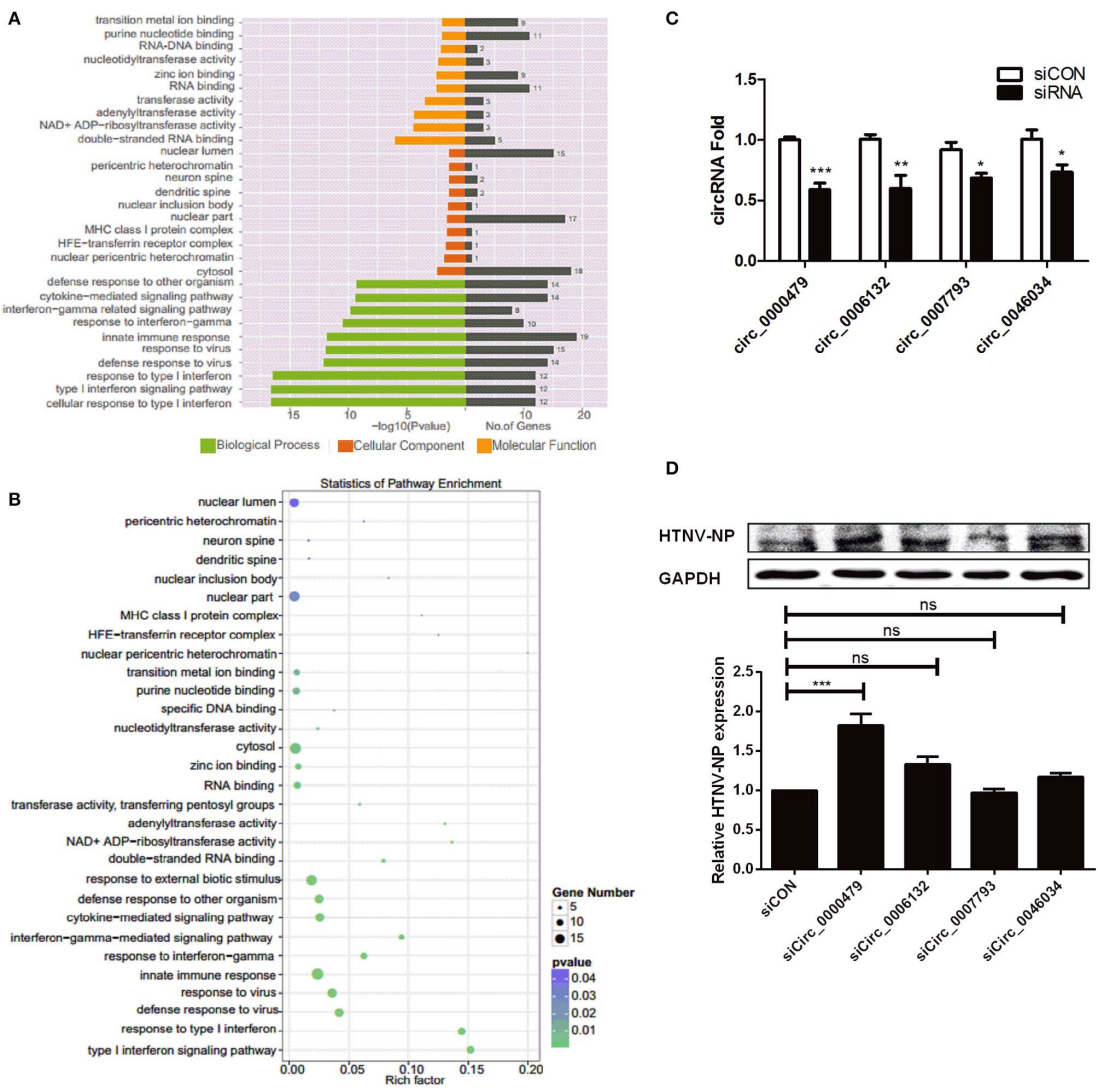
Verification of DE circRNAs after HTNV infection and exploration of their effect on HTNV infection. **(A)** GO functional enrichment analysis of parental genes of DE circRNAs. The *x*-axis shows the *P-*value and gene numbers, and the *y*-axis shows the GO term. **(B)** The 30 most enriched KEGG pathways based on hosting genes of dysregulated circRNA during HTNV infection. The *x*-axis shows the enrichment factor, and the *y*-axis shows the pathway names. The point size represents the number of genes enriched in a particular pathway. **(C)** The silencing efficiencies of siRNAs targeting the connected sites of circRNAs. Student's *t*-test, mean ± standard deviation (SD), *P < 0.05; **P < 0.01; ***P < 0.001. **(D)** Western blot assay of HTNV NP expression in HUVECs with knockdown of the circRNAs circ_0000479, circ_0006132, circ_0007793, and circ_0046034. One-way ANOVA with Dunnett's multiple comparison test. Mean ± SD, *P < 0.05; **P < 0.01; ***P < 0.001; ns, no significance. The experiments were performed at least three times independently.

To elucidate the biological function of DE circRNAs in HTNV infection, we examined the parental genes of DE circRNAs by functional analysis. Both GO and KEGG analysis were performed in this process. GO annotation analysis displayed that the parental genes of DE circRNAs were primarily enriched in diverse biological processes, including “cellular response to type I interferon,” “type I interferon signaling pathway,” “response to type I interferon,” and “defense response to virus” (Figure 2A). Moreover, the KEGG pathway enrichment analysis showed that plenty of the parental genes were principally enriched in “innate immune response,” “defense response to virus,” “cytokine-mediated signaling pathway,” and “response to virus” (Figure 2B). These data indicated that the parental genes of DE circRNAs are mainly involved in the host immune response to viral infection.

To gain insight into the effect of DE circRNAs on HTNV infection, we designed siRNAs specific for the head-tail junctions of various circRNAs, and transfected them into HUVECs. Knockdown efficiency was verified (Figure 2C). Then we found that knockdown of circ_0000479 could significantly increase the expression of HTNV NP (Figure 2D), suggesting circ_0000479 might be involved in the regulation of host-HTNV interplay. Nevertheless, the functional mechanisms of circRNAs remain to be further explored.

### Identification of DE mRNAs Between HTNV-Infected and Mock-Infected HUVECs

**Figure 3:**
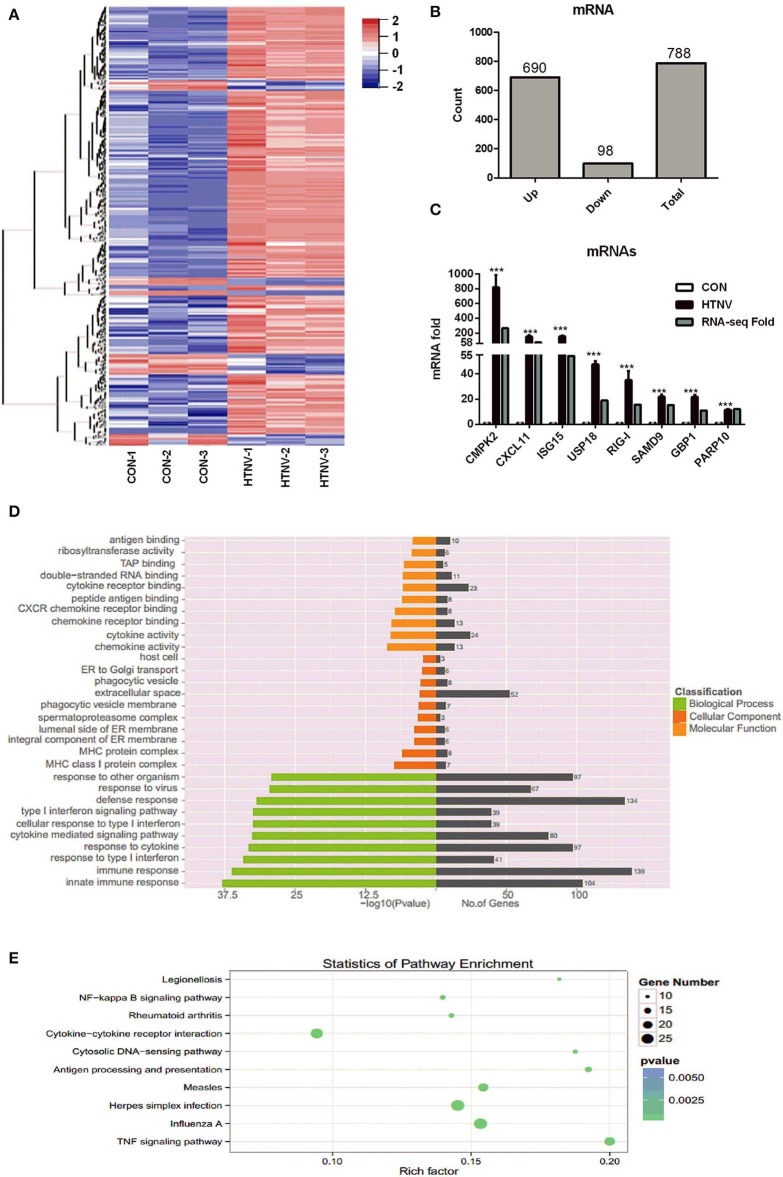
Identification of dysregulated mRNAs. **(A)** Heatmap and clustering analysis of DE mRNAs. Each row represents one mRNA and each column represents one sample; −2, −1, 0, 1, and 2 represent fold change. Red indicates high expression and blue represents low expression. CON-1, CON-2, and CON-3 represent three mock-infected samples; HTNV-1, HTNV-2, and HTNV-3 represent three HTNV-infected samples. **(B)** DE mRNAs count. **(C)** Verification of dysregulated mRNAs. HUVECs were infected with HTNV 76-118 for 3 days (MOI = 1). Then the total RNA was extracted and expression levels of the selected mRNAs were measured by RT-qPCR. Student's *t*-test, mean ± SD, ***P < 0.001. The experiment was performed at least three times independently. **(D)** GO functional enrichment analysis of DE genes. **(E)** Top 10 KEGG pathways of DE genes.

We analyzed the mRNA expression profiles in HTNV-infected and mock-infected HUVECs. We detected 788 DE mRNAs, i.e., 690 up-regulated and 98 down-regulated mRNAs (Figures 3A,B, Table S2). To verify the RNA-seq results, we randomly selected eight mRNAs (CMPK2, CXCL11, ISG15, USP18, RIG-I, SAMD9, GBP1, and PARP10) and measured their expression levels after HTNV infection by RT-qPCR (Figure 3C). They were all markedly up-regulated, in agreement with RNA-seq data. We next examined the functions of the DE mRNAs by GO analysis and KEGG pathway analysis. The top three significantly enriched GO terms were “innate immune response,” “immune response,” and “response to type I interferon” i.e., biological functions (Figure 3D). This implied that the cellular antiviral response was activated by HTNV infection. KEGG analysis showed that the enriched pathways were mainly involved in “TNF signaling pathway,” “Influenza A,” and “Herpes Simplex infection” (Figure 3E).

### Identification of DE miRNAs Between HTNV-Infected and Mock-Infected HUVECs

**Figure 4:**
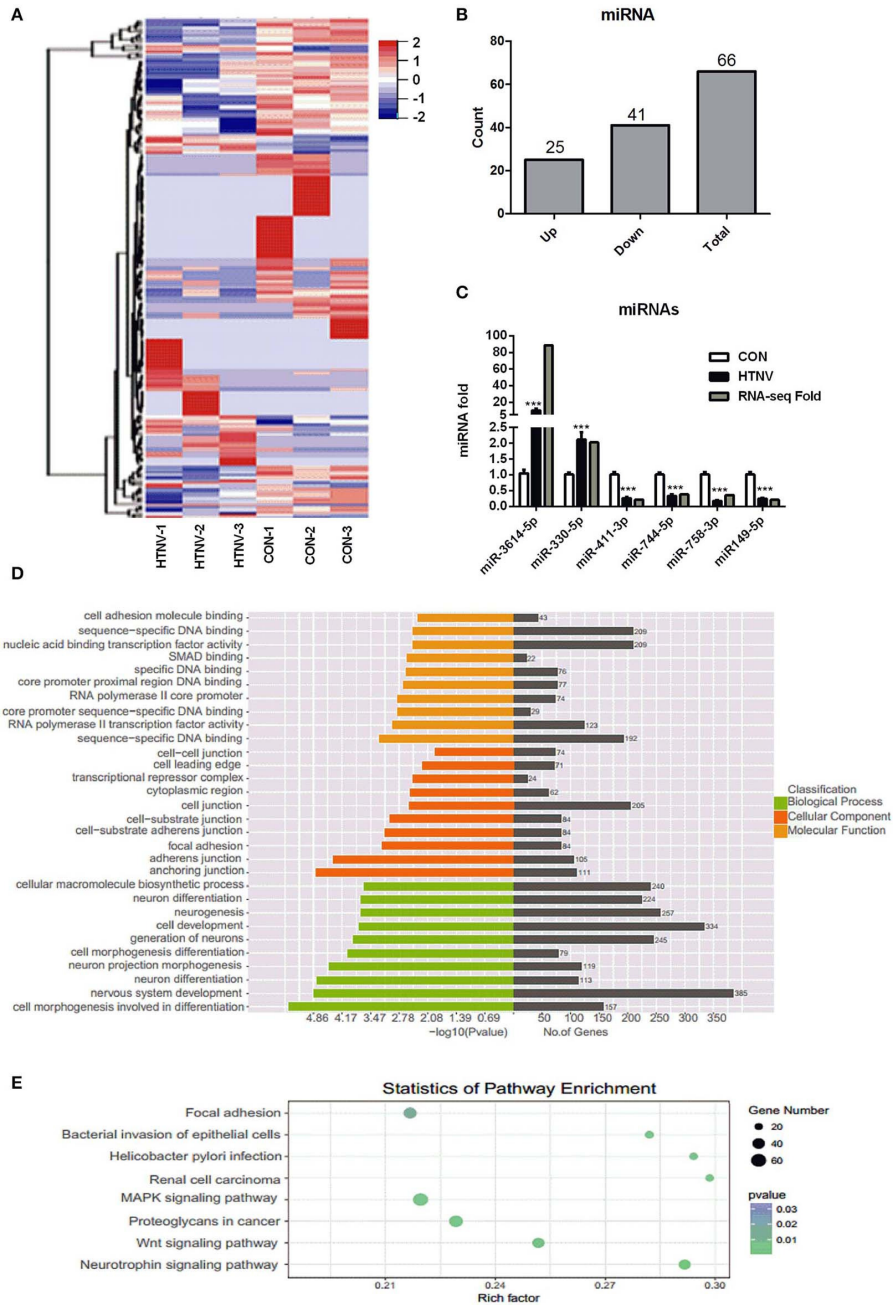
Identification of dysregulated miRNAs. **(A)** Heatmap and clustering analysis of DE miRNAs. Each row represents one miRNA and each column represents one sample; −2, −1, 0, 1, and 2 represent fold change. Red indicates high expression and blue represents low expression. CON-1, CON-2, and CON-3 represent three mock-infected samples; HTNV-1, HTNV-2, and HTNV-3 represent three HTNV-infected samples. **(B)** DE miRNAs count. **(C)** Verification of DE miRNAs upon HTNV infection. HUVECs were infected with HTNV 76-118 (MOI = 1) for 3 days. Then the total RNA was extracted and the expression levels of miRNAs were measured by RT-qPCR. Student's *t*-test, mean ± SD, ***P < 0.001. The experiment was performed at least three times independently. **(D)** GO functional enrichment analysis of target genes of DE miRNAs. The *x*-axis shows the *P*-value and gene numbers, and the *y*-axis shows the GO term. **(E)** KEGG pathway analysis revealed the top 10 enriched pathways of target genes of DE miRNAs.

To investigate the miRNA expression profiles of HTNV-infected and mock-infected HUVECs, six small RNA libraries were constructed and sequenced. Sequencing analysis revealed that a total of 66 miRNAs were significantly dysregulated upon HTNV infection; 25 were up-regulated and 41 were down-regulated (Figures 4A,B, Table S3). We verified the up- and down-regulation of six randomly selected DE miRNAs (up-regulated miRNAs: miR-3614-5p and miR-330-5p; down-regulated miRNAs: miR-411-3p, miR-744-5p, miR-758-3p, and miR-149-5p) by RT-qPCR. The RT-qPCR data were in agreement with RNA-seq data (Figure 4C). Next, we predicted the targets of the top 31 DE miRNAs; 2676 candidate target genes were obtained. GO analysis indicated that these target genes were mainly involved in “cell morphogenesis involved in differentiation,” “nervous system development,” and “neuron projection morphogenesis” (Figure 4D). Furthermore, the KEGG analysis manifested that the target genes of the DE miRNAs were mostly involved in “neurotrophin signaling pathway,” “Wnt signaling pathway,” “proteoglycans in cancer,” “MAPK signaling pathway,” and “renal cell carcinoma” (Figure 4E).

### Analysis of Regulatory Network of DE circRNAs, miRNAs, and mRNAs

**Figure 5:**
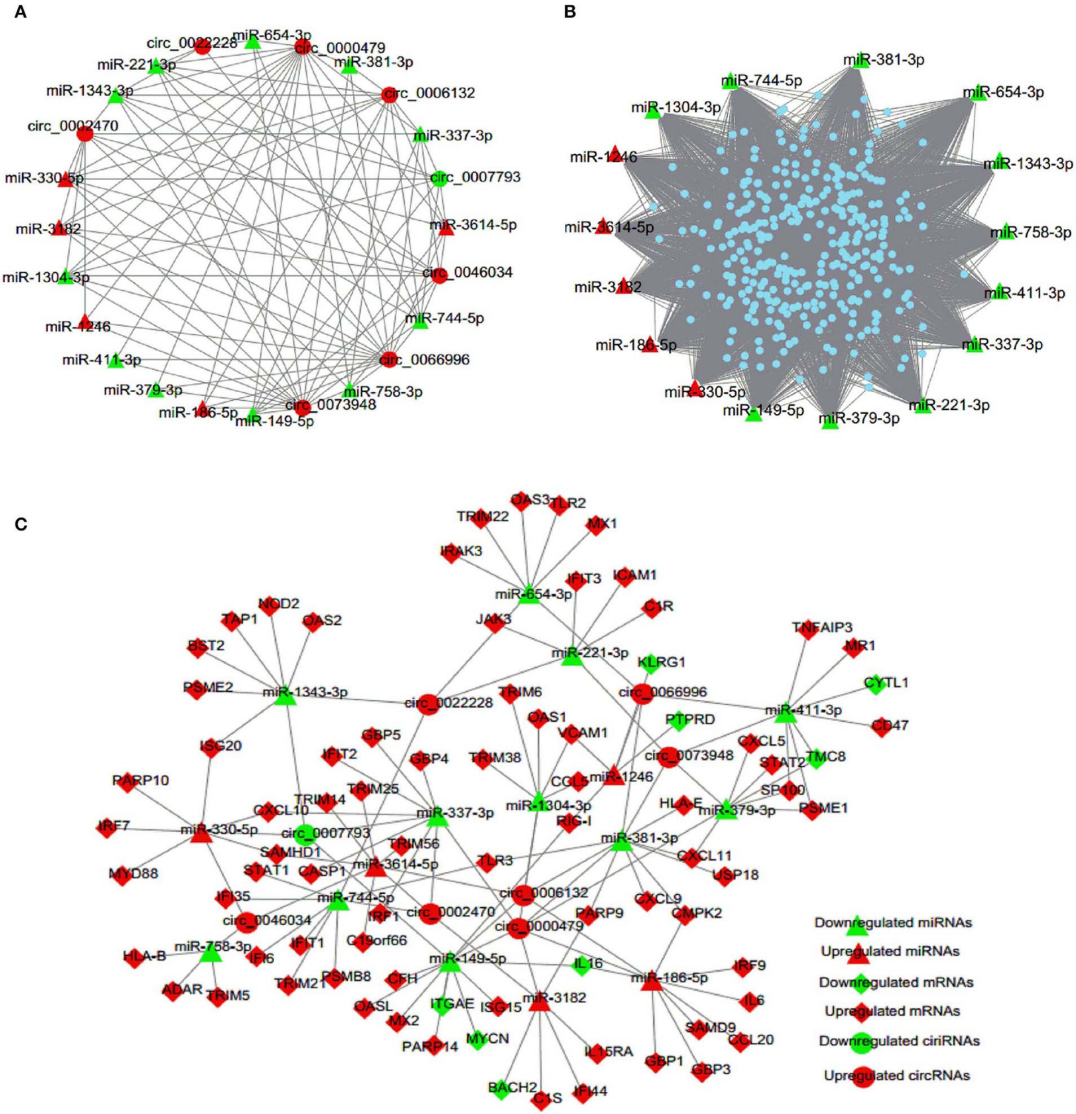
circRNA-miRNA-mRNA interaction network. **(A)** circRNA-miRNA regulatory network. Circles represent circRNA and triangles represent miRNA. **(B)** miRNA-mRNA regulatory network. Triangles represent miRNA and dots represent mRNA. **(C)** ceRNA co-expression network. Circles represent circRNA, triangles represent miRNA, and rhombi represent mRNA. Red and green represent up-regulated and down-regulated RNAs, respectively.

It was recently identified that circRNA can competitively sponge miRNAs, by which indirectly regulate gene expression (Wang M. et al., 2018; Mao et al., 2019; Rong et al., 2019). To examine the involvement of DE circRNAs, an integrative analysis of interplay between circRNAs and their target miRNAs was performed to elucidate their functional connections. The analysis of miRNA binding sites in circRNAs showed that 16 miRNAs, i.e., five up-regulated and 11 down-regulated miRNAs, had potential interactions with eight selected circRNAs (Figure 5A, Table S4). Then we predicted 320 target genes of these 16 DE miRNAs and constructed a miRNA-mRNA interaction network (Figure 5B, Table S5). Finally, in order to investigate the possibility that circRNAs act as ceRNAs in HTNV infection, we selected 80 enriched mRNAs that were involved in the innate immune pathway based on GO and KEGG analyses and constructed a ceRNA network through integrating the above circRNA-miRNA and miRNA-mRNA interaction networks (Figure 5C, Table S6). As shown in Figure 5C, circRNAs (circ_0000479, circ_0046034) may function as ceRNAs and sequester miR-149-5p to relieve its binding and targeting of mRNAs (RIG-I, IL16, MX2, etc.). RIG-I, also known as DDX58, was reported to inhibit HTNV replication by facilitating HTNV-induced IFN-β production (Ma et al., 2017). HTNV infection enhanced the production of multiple cytokines, including interleukin (IL) 6 (Jiang et al., 2008; Yu et al., 2014; Guo et al., 2017). Besides, we previously demonstrated that myxovirus resistance 2 (MX2, also known as MXB) served as a potential inhibitor in HTNV infection (Li et al., 2019). Taken together, the circRNAs/miRNAs could play important roles in HTNV-host interaction through regulating the expression of RIG-I, IL-6, and MXB associated with HTNV infection.

### Function of Dysregulated mRNAs and miRNAs in ceRNA Network Upon HTNV Infection

**Figure 6:**
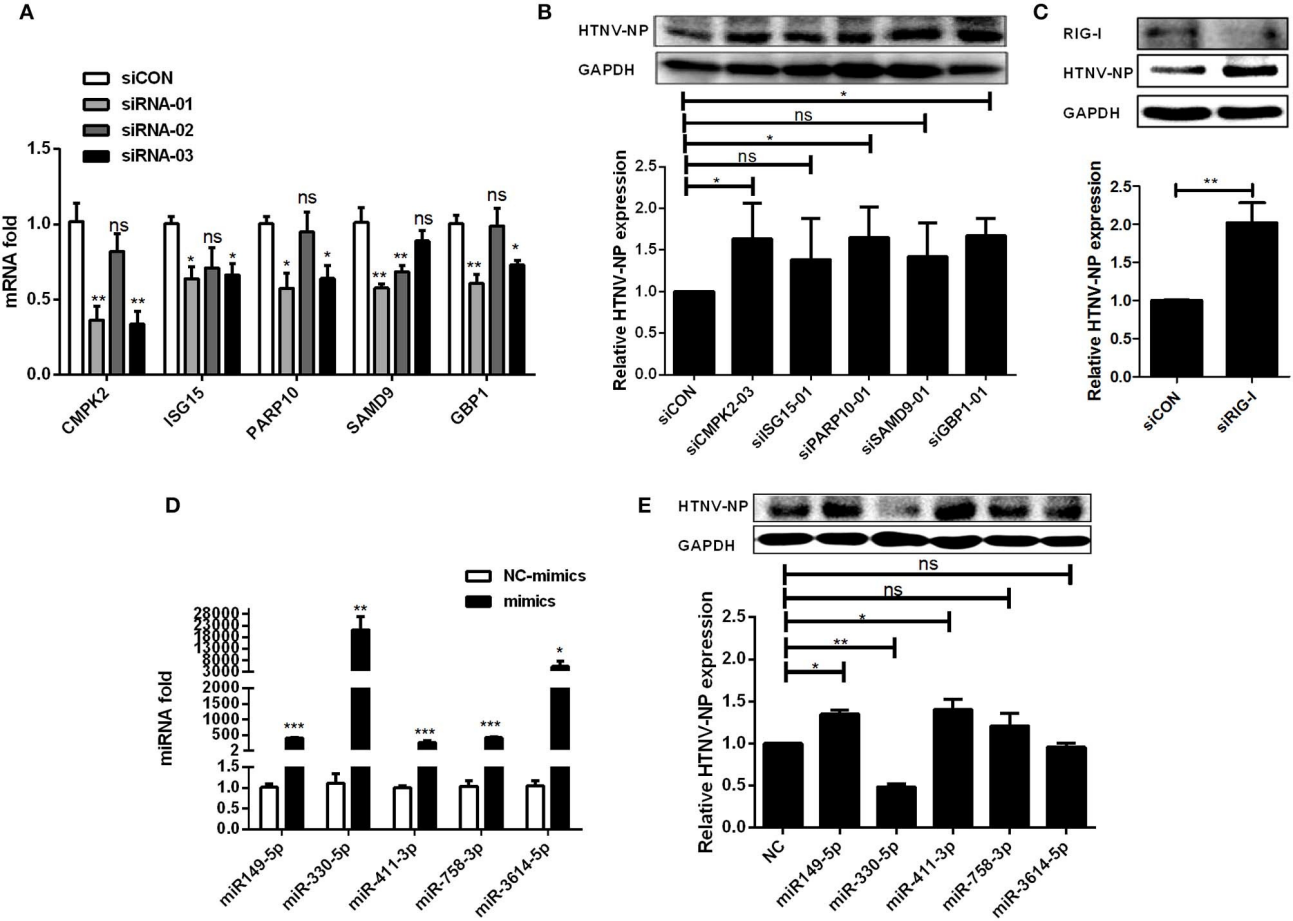
Exploration of the function of DE mRNAs and miRNAs upon HTNV infection in ceRNA network. **(A)** The interfering efficiencies of siRNAs targeting the different sites of mRNAs. **(B)** HUVECs were transfected with siCON or efficient siRNAs. 24 h after transfection, the cells were infected with HTNV at an MOI of 1 for 3 days. The expression of HTNV NP in HUVECs was measured by Western-blotting assay. **(C)** The expression of RIG-I and HTNV NP in HUVECs after interfering RIG-I and infected with HTNV. **(D)** The overexpression effect of miRNA mimics in HUVECs. **(E)** Western-blotting assay of HTNV NP expression in HUVECs transfected with miRNA mimics. Student's *t*-test, One-way ANOVA with Dunnett's multiple comparison test. Means ± SD, *P < 0.05; **P <0.01; ***P < 0.001; ns, no significance. The experiments were performed at least three times independently.

In order to figure out whether mRNAs and miRNAs in the ceRNA network have effects on viral infection, we investigated the effect of selected six representative mRNAs (CMPK2, ISG15, PARP10, SAMD9, GBP1, and RIG-I) involved in the ceRNA network upon HTNV infection. Firstly, we designed and transfected three specific siRNAs into HUVECs to decrease mRNA expression (Figure 6A). Then, we selected the most interference efficient siRNAs of each mRNA and transfected them into cells followed by HTNV infection to detect the expression levels of viral NP. The results showed that knockdown of CMPK2, PARP10, GBP1, and RIG-I could significantly increase the expression of HTNV NP (Figures 6B,C), suggesting these mRNAs could play a role in the cellular response against HTNV infection.

Meanwhile, to explore the role of DE miRNAs, we first transfected specific mimics of five representative miRNAs (miR-149-5p, miR-411-3p, miR-758-3p, miR-330-5p, and miR-3614-5p) into HUVECs and measured the miRNA expression levels by RT-qPCR (Figure 6D). After that, HUVECs were challenged with HTNV, and the expression level of viral NP was assessed. The data showed that miR-149-5p and miR-411-3p markedly increased the expression of HTNV NP, while miR-330-5p significantly inhibited viral replication (Figure 6E).

### Verification of ceRNA Network

**Figure 7:**
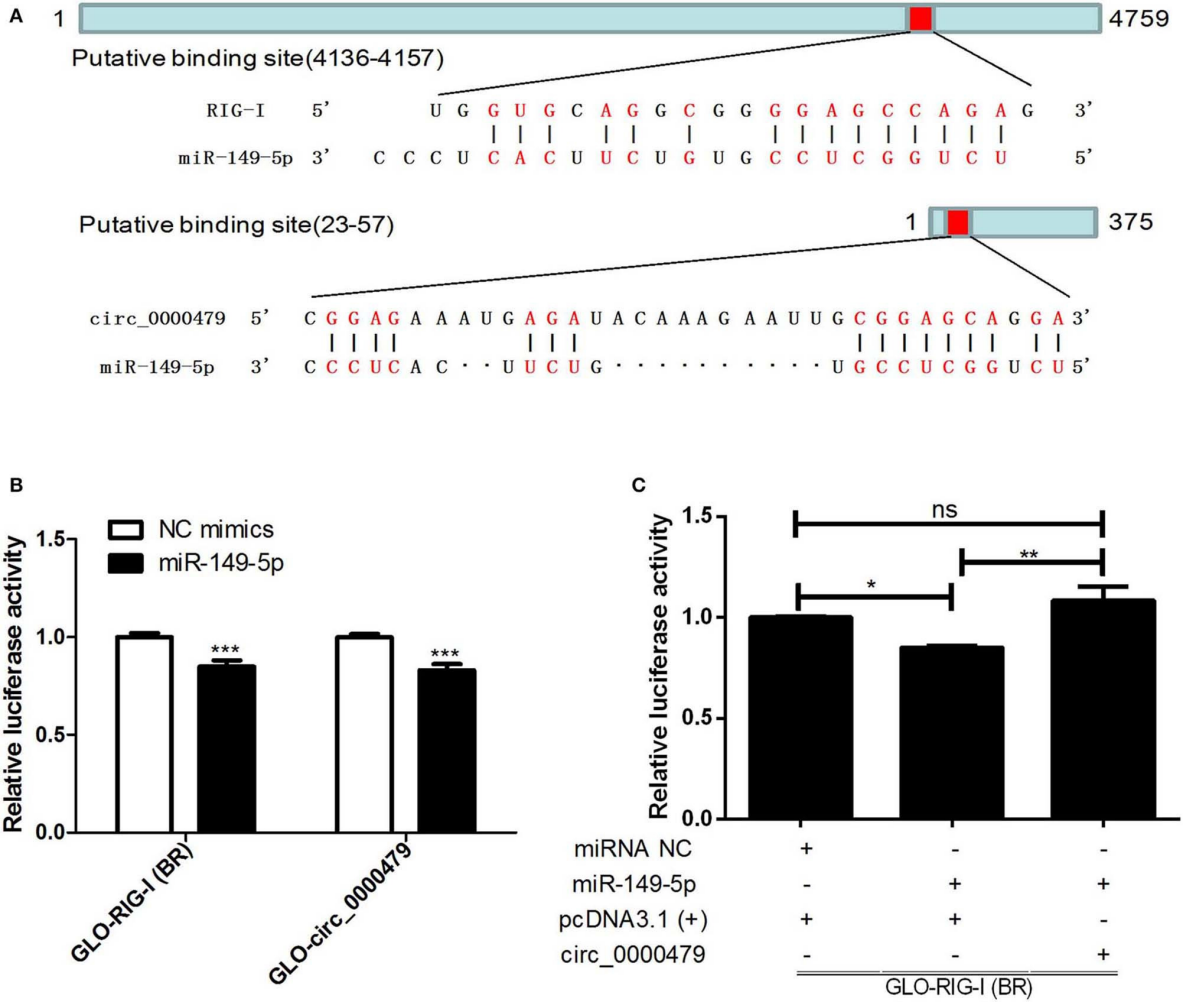
Verification of circRNA-miRNA-mRNA axis. **(A)** Putative binding sites of miR-149-5p to RIG-I and circ_0000479. **(B)** 293T cells were co-transfected with NC mimics or miR-149-5p and GLO plasmids harboring the RIG-I binding region (RIG-I-BR) and circ_0000479 for 48 h. Cells were lysed and then subjected to dual-luciferase reporter analysis. **(C)** 293T cells were co-transfected with miR-149-5p or negative control, GLO-RIG-I-BR, and circ_0000479 overexpressing plasmids. Cells were lysed and then subjected to dual-luciferase reporter analysis. Student's *t*-test, one-way ANOVA with Bonferroni's correction comparison test. Means ± SD, *P < 0.05; **P < 0.01; ***P < 0.001; ns, no significance. The experiments were performed at least three times independently.

We selected circ_0000479, RIG-I, and miR-149-5p from the ceRNA network, which have been verified to have a significant regulatory effect on HTNV replication based on the previous results, to verify the existence of the circRNA-miRNA-mRNA regulatory axis during viral infection by dual-luciferase reporter assay. The putative binding sites of miR-149-5p to RIG-I and circ_0000479 were first identified (Figure 7A). miR-149-5p significantly inhibited the luciferase activity of RIG-I-BR and circ_0000479 (Figure 7B). Furthermore, overexpression of circ_0000479 abrogated the inhibition of RIG-I-BR by miR-149-5p (Figure 7C). The results implicated that circ_0000479 can competitively bind miR-149-5p, thereby alleviating the inhibition of miR-149-5p on RIG-I. These results indicated that the complicated circRNA-miRNA-mRNA network is active during HTNV infection and suggested that these ceRNAs can not only regulate viral replication, but also constitute interactive networks among themselves.

## Discussion

The function of circRNAs has primarily been illustrated in cancer and disease progression (Greene et al., 2017; Zhang Y. et al., 2017; Qu et al., 2018). Nevertheless, the expression profiles, functions, and mechanisms of action of circRNAs in viral infection remain largely unknown. Several studies have addressed the identification, characterization, and function of circRNAs during viral infection (Shi et al., 2018; Wang S. et al., 2018; Zhang et al., 2018), but the properties and potential roles of circRNAs during HTNV infection have not been explored.

In the present study, we systematically analyzed circRNA, miRNA, and mRNA expression profiles in HTNV-infected HUVECs. We found that HTNV infection significantly impacts circRNA expression profiles. A total of 70 DE circRNAs were identified in HTNV-infected compared with mock-infected HUVECs. Of these, 65 circRNAs were up-regulated and five were down-regulated. The length, categories, and chromosomal distribution of DE circRNAs were similar to those observed in some other diseases (Huang et al., 2016; Chen J. et al., 2017; Zhang X. et al., 2017). We also identified 788 DE mRNAs and 66 DE miRNAs, i.e., 690 up-regulated mRNAs, 98 down-regulated mRNAs, 25 up-regulated miRNAs, and 41 down-regulated miRNAs, upon HTNV infection. Interestingly, most DE mRNAs (88%) and DE circRNAs (93%) were up-regulated, while most DE miRNAs (62%) were down-regulated. These results indicated that the regulatory mechanism of miRNAs in HTNV-cell interaction could be different from that of circRNAs and mRNAs. It also supports the theory that a complex regulatory ceRNA network exists.

As is well-known, innate antiviral and inflammatory responses can immediately be elicited upon viral infection. These responses must be regulated delicately to prevent viral dissemination and present appropriate effective immune responses. Several researches reported that hantaviruses infection is absence of visible cytopathic effects (CPE), and concluded that HFRS might be caused by unbalanced and inappropriate immune responses elicited by hantaviruses (Pensiero et al., 1992; Vapalahti et al., 2001). Consistently, GO enrichment analysis and KEGG pathway analysis for annotating the parental genes of the DE circRNAs revealed that they were mostly involved in the host immune response, including “innate immune response,” “response to virus,” “defense response to other organism,” “cytokine-mediated signaling pathway,” “response to type I interferon,” “type I interferon signaling pathway,” and “cellular response to type I interferon,” indicating that DE circRNAs may regulate HTNV infection through host immune signaling pathways and antiviral responses, contributing to viral pathogenic progression.

GO analysis and KEGG pathway analysis were also carried out to annotate the DE mRNAs upon HTNV infection. In line with the above data, the enrichment analyses revealed that the HUVECs initiated a strong antiviral defense upon HTNV infection, as observed with other viral infections such as Influenza A virus and Herpes Simplex Virus. GO enrichment analysis and KEGG analysis of mRNAs targeted by the identified miRNAs showed that predicted target genes are involved in biological processes such as cell development, cell morphogenesis differentiation, cellular macromolecule biosynthesis, the MAPK signaling pathway, and the Wnt signaling pathway, which have previously been documented to regulate cell fate and behavior. This suggests that DE miRNAs may be the principal regulators of cellular function during HTNV infection.

More and more studies have identified that circRNAs could serve as miRNA sponges, indirectly regulating gene expression. Therefore, to uncover the detailed function and mechanism of DE circRNAs in HTNV infection, we constructed a circRNA-miRNA-mRNA ceRNA network. The integrative analysis of circRNA-associated ceRNAs demonstrated the regulatory functions of circRNAs and specific interplay with other RNAs by circRNA-miRNA-mRNA regulatory axis during the cellular innate immune response against HTNV. Through ceRNA analysis, we found at least three regulatory circRNA-miRNA-mRNA axes potentially involved in the regulation of HTNV, i.e., circ_0002470-miR-3182-IFI44, circ_0006132-miR-1304-3p-OAS1, and circ_0000479-miR-337-3p-CASP1. It has been reported that IFI44 is an IFN-inducible protein, related to infection of several viruses (Balan et al., 2006; Power et al., 2015; deDiego et al., 2019). Other studies documented that OAS1 plays an important role in antiviral activity as antiviral factor (Knapp et al., 2003; Melchjorsen et al., 2009; Singh et al., 2016). CASP1 participates in the composition of the inflammasome which actives the pro-inflammatory cytokines IL-18 and IL-1β, resulting in the proinflammatory cell death known as pyroptosis (Lupfer et al., 2015). Recently, it has been confirmed that the NLRP3 inflammasome is responsible for hantavirus-induced expression of IL-1β (Ye et al., 2015). Furthermore, antiviral and proinflammatory responses to hantavirus infection play important roles in regulating host defense and disease manifestation (Shin et al., 2014). Therefore, we speculate that dysregulation of the identified DE circRNAs might be related to HTNV replication by antagonizing the inhibition of miRNAs on mRNAs.

Moreover, we further explored the effects of six DE mRNAs, four DE circRNAs, and five DE miRNAs on viral replication. The results showed that knockdown of mRNAs (CMPK2, PARP10, GBP1, and RIG-I) and circ_0000479 could dramatically increase HTNV NP expression, suggesting that these RNAs are potential inhibitors of HTNV infection. While overexpression of miR-149-5p and miR-411-3p can facilitate viral replication; miR-330-5p can markedly inhibit viral replication. To date, there were neither characteristics nor function reports about circ_0000479. While in the ceRNA network, we predicted that circ_0000479 could sponge miR-149-5p to regulate the expression of genes, such as RIG-I, IL16, ISG15, ITGAE, MX2, MYCN, OASL, PARP14, CFH, which were functional in innate immune pathways. Consequently, these correlations preliminarily explained inhibition mechanism of circ_0000479 on HTNV replication.

We further verified one of circRNA-associated regulatory axis by luciferase reporter assay. the data showed that miR-149-5p can target RIG-I and circ_0000479, and circ_0000479 could regulate the expression of RIG-I by sponging miR-149-5p. Here, for the first time, we identified and confirmed the circ_0000479-miR-149-5p-RIG-I regulatory axis in HTNV infection. As is well-known, the pattern recognition receptor RIG-I initiates the antiviral immune response and increases type I interferon expression after recognition of RNA viruses (Leung, 2019). In agreement with these observations, inhibition of RIG-I promotes HTNV replication (Ma et al., 2017). The data obtained in the present study will enrich our understanding of the interaction between HTNV and host cells. However, other circRNA-miRNA-mRNA axes in ceRNA network deserve verification and clarification. Further research on the role of ncRNAs in HTNV infection is warranted.

In conclusion, this study is the first to identify a circRNA expression profile and construct a circRNA-related regulatory network involved in interaction between HTNV and host cells, in particular in the host defense to viral infection (as shown in Graphical Abstract). This study therefore provides novel insightfor illuminating the pathogenesis of HTNV.

## Data Availability Statement

The datasets generated for this study can be found in the Gene Expression Omnibus with accession nos. GSE133634, GSE133751, GSE133319.

## Author Contributions

SL, FL, and WH conceived and designed the experiments. NZ and WH contributed to the funding acquisition. SL, NZ, WG, XWa, KL, JY, CJ, SH, HXia, and XWu performed the experiments. SL, YL, HXio, LC, ZG, and FL collected and analyzed the data. SL wrote original draft. FL and WH revised the paper. All authors approved the final version of the manuscript.

### Conflict of Interest

The authors declare that the research was conducted in the absence of any commercial or financial relationships that could be construed as a potential conflict of interest.
